# Supplementary material for: Direct observation of independently moving replisomes in Escherichia coli
Source: Nat Commun. 2020 Jun 19;11:3109. doi: 10.1038/s41467-020-16946-7 (PMC7305307; doi:10.1038/s41467-020-16946-7)
Supplement: Supplementary file 3 — Description of Additional Supplementary Files [file 41467_2020_16946_MOESM3_ESM.pdf]

Description of Additional Supplementary Files

File Name: Supplementary Movie 1
Description: Timelapse video of cell elongation

File Name: Supplementary Movie 2
Description: Timelapse video of replisome splitting in elongated cells

File Name: Supplementary Movie 3
Description: Timelapse video of widened E.coli cell division

File Name: Supplementary Movie 4
Description: Timelapse video of chromosome replication in widenend E.coli cell
