# Supplementary material for: Direct observation of independently moving replisomes in Escherichia coli
Source: Nat Commun. 2020 Jun 19;11:3109. doi: 10.1038/s41467-020-16946-7 (PMC7305307; doi:10.1038/s41467-020-16946-7)
Supplement: Supplementary file 8 — Reporting Summary [file 41467_2020_16946_MOESM8_ESM.pdf]

nature research

Corresponding author(s): Cees Dekker

Last updated by author(s): May 18, 2020

Reporting Summary

Nature Research wishes to improve the reproducibility of the work that we publish. This form provides structure for consistency and transparency in reporting. For further information on Nature Research policies, see Authors & Referees and the Editorial Policy Checklist.

Statistics

For all statistical analyses, confirm that the following items are present in the figure legend, table legend, main text, or Methods section.

n/a	Confirmed	
☐	☒	The exact sample size (n) for each experimental group/condition, given as a discrete number and unit of measurement
☐	☒	A statement on whether measurements were taken from distinct samples or whether the same sample was measured repeatedly
☒	☐	The statistical test(s) used AND whether they are one- or two-sided<br>*Only common tests should be described solely by name; describe more complex techniques in the Methods section.*
☒	☐	A description of all covariates tested
☒	☐	A description of any assumptions or corrections, such as tests of normality and adjustment for multiple comparisons
☐	☒	A full description of the statistical parameters including central tendency (e.g. means) or other basic estimates (e.g. regression coefficient) AND variation (e.g. standard deviation) or associated estimates of uncertainty (e.g. confidence intervals)
☒	☐	For null hypothesis testing, the test statistic (e.g. F, t, r) with confidence intervals, effect sizes, degrees of freedom and P value noted<br>*Give P values as exact values whenever suitable.*
☒	☐	For Bayesian analysis, information on the choice of priors and Markov chain Monte Carlo settings
☒	☐	For hierarchical and complex designs, identification of the appropriate level for tests and full reporting of outcomes
☒	☐	Estimates of effect sizes (e.g. Cohen's d, Pearson's r), indicating how they were calculated

Our web collection on statistics for biologists contains articles on many of the points above.

Software and code

Policy information about availability of computer code

Data collection	NIS-Elements Imaging Software (ver. 4.30.02), Nikon Instruments Inc., United States
Data analysis	Huygens Professional, (ver.19.04) Scientific Volume Imaging, Hilversum, The Netherlands<br>MATLAB (2019b), The MathWorks, Inc., United States.

For manuscripts utilizing custom algorithms or software that are central to the research but not yet described in published literature, software must be made available to editors/reviewers. We strongly encourage code deposition in a community repository (e.g. GitHub). See the Nature Research guidelines for submitting code & software for further information.

Data

Policy information about availability of data

All manuscripts must include a data availability statement. This statement should provide the following information, where applicable:

- Accession codes, unique identifiers, or web links for publicly available datasets
- A list of figures that have associated raw data
- A description of any restrictions on data availability

The source data underlying Figs 2e &2h are provided as a Source Data file. The raw microscopy data supporting the findings of this manuscript (Figure 2, Figure 3, Figure 4, and Supplementary Figures 1,2,5,6,7,10,11,13,14,16 and 17) are freely available for download from 4TU.ResearchData repository [https://doi.org/10.4121/uuid:23de478d-8d08-4149-abd6-3398cbe14847].

Field-specific reporting

Please select the one below that is the best fit for your research. If you are not sure, read the appropriate sections before making your selection.

☒ Life sciences ☐ Behavioural & social sciences ☐ Ecological, evolutionary & environmental sciences

For a reference copy of the document with all sections, see nature.com/documents/nr-reporting-summary-flat.pdf

Life sciences study design

All studies must disclose on these points even when the disclosure is negative.

Sample size	Typically between several hundred cells were selected for the analysis. The number was chosen at random, depending on the density of the number of cells per image position. Typically up to few hundred cells are reported in similar replication studies.
Data exclusions	If the cells were dead, they were excluded from the statistical analysis. The total number of dying cells was typically ~1% in all experiments.
Replication	To ensure reproducibility, all samples were repeated at least in biological duplicates or triplicates. All experiments were successful.
Randomization	Samples were allocated to experimental groups based on the growth conditions (growth temperature, growth media, presence of antibiotics etc.) used. No randomization was not relevant.
Blinding	Blinding was not performed, because depending on the growth conditions (growth temperature, growth media, presence of antibiotics) the cell morphology was clearly different between cells, making the blinding not possible.

Reporting for specific materials, systems and methods

We require information from authors about some types of materials, experimental systems and methods used in many studies. Here, indicate whether each material, system or method listed is relevant to your study. If you are not sure if a list item applies to your research, read the appropriate section before selecting a response.

Materials & experimental systems

n/a	Involved in the study	
☒	☐	Antibodies
☒	☐	Eukaryotic cell lines
☒	☐	Palaeontology
☒	☐	Animals and other organisms
☒	☐	Human research participants
☒	☐	Clinical data

Methods

n/a	Involved in the study	
☒	☐	ChIP-seq
☒	☐	Flow cytometry
☒	☐	MRI-based neuroimaging
